# Recent progress in mapping the emerging landscape of the small-cell lung cancer genome

**DOI:** 10.1038/s12276-019-0349-5

**Published:** 2019-12-12

**Authors:** Kee-Beom Kim, Colin T. Dunn, Kwon-Sik Park

**Affiliations:** 1 Departments of Microbiology, Immunology, and Cancer Biology, University of Virginia School of Medicine, Charlottesville, VA 22908 USA; 2 School of Medicine MD Program, Case Western Reserve University, Cleveland, OH 44106 USA

**Keywords:** Cancer epigenetics, Diseases

## Abstract

Small-cell lung cancer (SCLC) remains the deadliest of all the lung cancer types. Its high mortality is largely attributed to the invariable development of resistance to standard chemo/radiotherapies, which have remained unchanged for the past 30 years, underscoring the need for new therapeutic approaches. The discovery of molecular targets for chemoprevention and treatment has been hampered by the poor understanding of SCLC progression. In recent years, comprehensive omics-based analyses have led to the discovery of recurrent alterations in patient tumors, and functional studies using genetically engineered mouse models and patient-derived tumor models have provided information about the alterations critical for SCLC pathogenesis. Defining the somatic alterations scattered throughout the SCLC genome will help to understand the underlying mechanism of this devastating disease and pave the way for the discovery of therapeutic vulnerabilities associated with the genomic alterations.

## Small cell lung cancer: Genome insights for improving treatment

Alterations in the small cell lung cancer (SCLC) genome are critical for disease progression and relapse. A complete map of the genome in cancerous cells would greatly improve the chances of successfully treating this deadly disease. SCLC is often detected too late, and only five per cent of patients survive beyond five years after diagnosis. While the disease initially responds to standard chemotherapy, the cancer cells quickly build resistance and relapse follows. Kwon-Sik Park at the University of Virginia, Charlottesville, US, and co-workers reviewed current understanding of SCLC genome alterations. The latest research highlights substantial variations in the SCLC genome between patients, with implications for existing treatment regimens. Researchers have made considerable progress in profiling the genome, with significant alterations, mutations and potential therapeutic targets now being explored in genetically engineered mouse models and patient-derived tumor models.

## Introduction

Lung cancer is the leading cause of cancer deaths among both men and women. The American Cancer Society estimates that there were more than 140,000 deaths from lung cancer in 2019 in the United States alone. Lung cancer is largely divided into histological types, including non-small-cell lung cancer (NSCLC), small-cell lung cancer (SCLC), and lung carcinoid cancer^1^. NSCLC accounts for ~85% of all lung cancer diagnoses and comprises several subtypes, including lung adenocarcinoma, squamous cell carcinoma, and large cell carcinoma. SCLC accounts for ~10 to 15% of all lung cancers and is a high-grade neuroendocrine tumor that is distinct from rare subtypes of large-cell neuroendocrine carcinoma and neuroendocrine carcinoid^2–4^. While the prognosis for most lung cancer types is generally poor, SCLC is the deadliest, being uniformly fatal and having a 5-year survival of ~5%^5^. The major contributing factors to the poor outcomes seen in SCLC are difficulties in early detection and the inadequacy of current therapy. SCLC is often detected in late stages, which limits treatment options to cytotoxic chemotherapies, usually a combination of platinum-based alkylating agents (e.g., cisplatin/carboplatin) and topoisomerase inhibitors (e.g., irinotecan/etoposide). These chemotherapy regimens have remained largely unchanged for the past 40 years and fail to significantly improve overall patient survival. Most SCLC tumors initially respond well to standard chemo/radiotherapy but invariably relapse with the development of chemoresistance. Few secondary therapies, including topotecan and cyclophosphamide, maintain a durable response. Therefore, a better understanding of the mechanism underlying the development of resistance may lead to the development of a novel therapeutic strategy. In addition, an improved understanding of the mechanisms of tumor initiation and early-stage progression may facilitate the development of novel means for early detection and prevention. To these ends, extensive research efforts have been focused on defining the molecular determinants of SCLC. SCLC has long been viewed as a homogeneous disease characterized by a set of common pathological features, including a distinct morphology of small cells with scant cytoplasm and ill-defined borders, rapid growth with a high proliferation rate and accompanying necrosis, and near universal loss of both the *RB1* (encoding RB) and *TP53* (encoding p53) genes. The perceived homogeneity of SCLC has been reflected in clinical practice, as most SCLC patients receive identical chemotherapy. Highly proliferative tumors such as SCLC are more sensitive to these DNA-damaging drugs and undergo cell death. However, a growing body of evidence from molecular analyses of patient samples and genetically defined models indicates considerable heterogeneity in the histology, cell morphology, degree of neuroendocrine differentiation, and role of neuronal lineage-specific transcription factors in this disease. Integration of these aspects of heterogeneity has led to a model of SCLC subtypes, namely, SCLC-A (ASCL1-positive), SCLC-N (NEUROD1-positive), SCLC-P (POU2F3-positive), and SCLC-Y (YAP1-positive); SCLC-A and SCLC-N are neuroendocrine subtypes, whereas SCLC-P and SCLC-Y are nonneuroendocrine subtypes^6^. Importantly, these subtypes can be linked to specific biomarkers that are either targets of specific drugs or predictors of drug response, for example, DLL3 (a membrane target for the antibody-drug conjugate Rova-T) in SCLC-A and AURKA (a kinase target for alisertib) in SCLC-N^7,8^. The heterogeneity in SCLC was first noted years ago by Carney et al., who described the ‘variant’ form of cells with c-MYC amplification, partial or complete loss of neuroendocrine differentiation, and partial epithelial-to-mesenchymal transition phenotype, as opposed to the ‘classic’ sphere/aggregate-forming neuroendocrine cells^9^. While the current characterization by molecular subtypes does not integrate information from the SCLC genome, functional interrogation of recurrent genomic alterations, as well as expansion of the dataset will lead to a robust genotype-based classification that may inform subtype-specific treatment.

## Profiles of the SCLC genome

### Copy number alterations

Array-based comparative genomic hybridization (aCGH) and array-based SNP (single-nucleotide polymorphism) analysis drastically increase the resolution of somatic copy number alterations from the chromosome level to the level of a single gene (Table 1). These analyses confirmed recurrent losses in the 3p and 17p regions, harboring *FHIT*, *RASSF1*, and *TP53*, and losses in the 13q and 10q regions, harboring *RB1* and *PTEN*^10–16^. Amplification of the 1p, 2p, and 8q regions, harboring MYCL (L-MYC), MYCN (N-MYC), and MYC (c-MYC) was also noted^17–19^. Amplification of MYC family genes accounts for up to 50% of all SCLC cases. These amplifications are largely mutually exclusive, consistent with the idea of functional redundancy among the MYC family genes in their contribution to SCLC. Recent whole-exome sequencing studies identified previously unknown copy number alterations, including focal loss of *SLIT2* (encoding a ligand for ROBO1) and focal amplification of *CCNE1, SOX2*, *FGFR1*, *IRS2*, and *NFIB*^20–22^. The genes encoding SOX proteins, particularly SOX2, are amplified in a significant portion of patient tumors^22^. Given the roles of these SOX proteins in the reprogramming of somatic cells into a stem/progenitor cell phenotype and in regulating lung progenitor cells^23,24^, tumor cells may coopt these proteins to promote self-renewal and dedifferentiation. *FGFR1* and *IRS2* amplifications indicate deregulation of receptor kinase signaling in a subset of tumors, raising the prospect of targeting this molecular subgroup with specific tyrosine kinase inhibitors*. NFIB* encodes a member of the nuclear factor I (NFI) family of transcription factors that play important roles in lung and brain development by regulating the expression of a wide spectrum of genes^25,26^. While *NFIB* amplification is infrequently detected in primary tumors, this gene is often overexpressed and amplified in SCLC cell lines (34%) that were mostly derived from metastatic tumors^21,27,28^. These observations suggest that increased activity of this transcription factor could promote both tumor development and metastasis.

**Table 1:** List of genes with copy number alterations in SCLC.

Gene	Amplification/deletion	Functional validation	Study			
			George^20^	Peifer^21^	Rudin^22^	Augert^39^
*TP53*	Deletion	^29^	O			O
*RB1*	Deletion	^29^	O			O
*CDKN2A*	Deletion	nd	O			
*FHIT*	Deletion	nd	O	O	O	
*RASSF1A*	Deletion	^111^			O	
*MYC*	Amplification	^7^	O	O		O
*MYCL*	Amplification	^90^	O	O	O	
*MYCN*	Amplification	nd	O	O	O	
*CCNE1*	Amplification	nd		O		O
*MET*	Amplification	^112,113^				
*FGFR1*	Amplification	nd	O			O
*IRS2*	Amplification	nd	O			
*NFIB*	Amplification	^27,103–105^		O		
*SOX2*	Amplification	^22^			O	
*SOX4*	Amplification	nd			O	

This table lists genes that have been found deleted or amplified in multiple studies, including the four different studies indicated above. *MET* and *NFIB* amplifications were found in other studies listed in the main text. The numbers in the column ‘Functional validation’ are references. nd: not determined

### High mutational rates

**Fig. 1:**
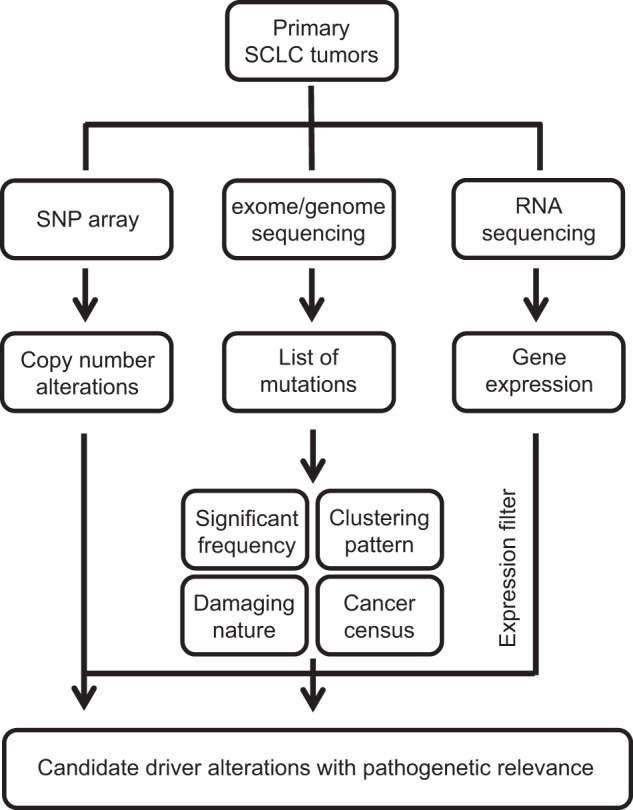
Schematic of the integrative approach to identify pathogenetically relevant alterations. This schematic, modified from George et al.^20^, illustrates the process of identifying alterations with a high likelihood of pathological relevance. Candidate alterations extracted from sequencing results are filtered for significant frequency, clustering pattern, damaging nature, and cancer census and further examined for the presence of coherent copy number alterations and run through expression filters (e.g., gene expression or change). Candidate drivers can be directly identified from the analysis of copy number alterations using single nucleotide polymorphism (SNP) arrays or from chimeric transcripts due to gene fusion.

**Fig. 2:**
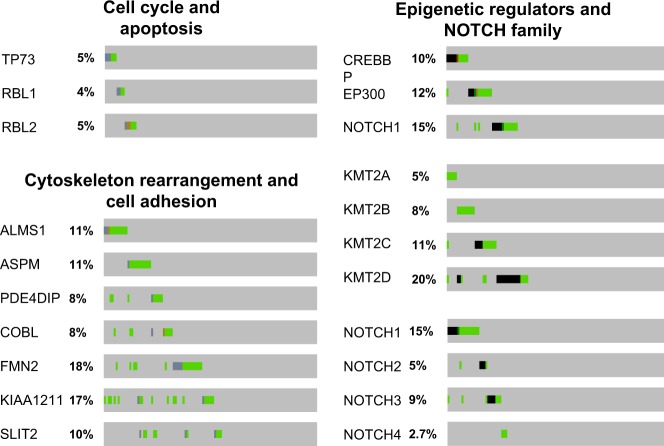
Graphical summaries of mutations in selected sets of genes. Each oncoprint, obtained from cBioportal, is a graphical summary of mutations in the potentially related genes across 110 SCLC patient tumors^20^. Although not statistically significant, there are trends toward mutual exclusivity among mutations in *TP73*, *RBL1*, and *RBL2*; *CREBBP*, *EP300*, and *NOTCH1*; and *ALMS1* and *ASPM*.

A major breakthrough in profiling the SCLC genome came when Peifer et al., Rudin et al., and George et al. provided the first overview of the genomic landscape of SCLC, identifying a large number of nonsynonymous (changing amino acid sequence) mutations at a rate of 8 per million nucleotides on average^20–22^. This extremely high mutational rate is attributed to the well-known association of SCLC patients with heavy smoking; indeed, the tobacco exposure signature (C:G > A:T transversion) was found in a significant portion (28%) of all mutations^20^. The other most notable alteration is biallelic loss-of-function alterations in both *RB1* and *TP53* in nearly all SCLC tumors, supporting the long-standing concept of loss of tumor suppressor activity as the rate-limiting event for SCLC initiation, which was validated in the genetically engineered mouse models^29^. However, the abundance and heterogeneity of mutations of unknown significance present a daunting challenge to defining the cancer genome and gaining mechanistic insight into the pathophysiology of SCLC. To identify pathogenetically relevant mutations, these genomics studies applied analytical filters including ‘significant occurrence’ (mutation rates higher than expected with a *q-*value < 0.05 after correction for gene expression); ‘clustering pattern’ (enrichment of mutations in DNA sequences encoding protein domains functionally related to tumor suppressor or oncogenic functions); ‘damaging nature of mutations’; and ‘cancer census’ (lists of genes frequently affected by somatic alterations in human cancers, for example, the Cancer Gene Census and COSMIC databases) (Fig. 1). A list of mutations filtered through these criteria and accounting for expression in neuroendocrine cells is compiled in Table 2. The relatively limited number of tumor samples precludes a robust analysis of functional relationships among the filtered alterations based on mutual exclusivity and cooccurrence. Nonetheless, mutations in *CREBBP*, *EP300*, *TP73*, *RBL1*, *RBL2*, and *NOTCH* family genes appeared largely mutually exclusive (Fig. 2), suggesting a common pathway affected by inactivation of these genes. Alternatively, gene ontology may lead to classification of a majority of the mutated genes in SCLC into the following groups: regulators of cell cycle and death, epigenetic regulators, receptor tyrosine kinases, and regulators of cytoskeleton dynamics and cell adhesion.

**Table 2:** List of genes with recurrent mutations in SCLC.

Gene	Frequency (%)	Functional validation	Study			
			George^20^	Peifer^21^	Rudin^22^	Augert^39^
Cell cycle and apoptosis						
* TP53*	79–98	^29^	O	O	O	O
* RB1*	35–91	^29^	O	O	O	O
* RBL1*	3-4	nd	O	O		O
* RBL2*	5–7	^89^	O	O		
* TP73*	2–7	^114^	O	O	O	O
NOTCH pathway						
* NOTCH1*	2–15	^20,107^	O	O	O	O
* NOTCH2*	4-5	^20,107^	O		O	O
* NOTCH3*	4–9	^115^	O	O	O	O
* NOTCH4*	2.7–10	nd	O	O		O
Epigenetic regulators						
* CREBBP*	4–14	^55^	O	O	O	O
* EP300*	7–12	nd	O	O	O	O
* KMT2A*	5–10	nd	O	O		O
* KMT2B*	8	nd	O			
* KMT2C*	7–11	nd	O	O		O
* KMT2D*	6–27	nd	O		O	O
* KDM6A*	2.7–4	nd	O	O	O	O
* SETD2*	2.7–7	nd	O			O
* PBRM1*	0.9–7	nd	O		O	O
* ARID1A*	3-4	nd	O		O	O
* ARID1B*	4–10	nd	O	O		O
* CHD7*	10	nd	O			O
Regulators of cytoskeleton and cell adhesion						
* ALMS1*	8–17	nd	O	O	O	O
* ASPM*	6–14	nd	O	O	O	O
* PDE4DIP*	6–8	nd	O	O	O	O
* COBL*	5–10	nd	O	O	O	O
* FMN2*	7–18	nd	O	O	O	O
* KIAA1211*	3–17	nd	O	O	O	O
* COL4A2*	10	nd			O	O
* COL22A1*	18–21	nd	O	O	O	O
* SLIT2*	4–17	nd	O	O	O	O
Kinase signaling						
* PIK3CA*	2.7–6	nd	O		O	O
* PTEN*	4–14	^87,88^	O	O	O	O
* EPHA7*	4–10	nd	O	O	O	O

This table lists genes that have been found mutated in the four different studies, as indicated. These genes are listed from top to bottom and have functionally related genes in proximity. The far-left column indicates the functions or pathways that the genes are related to. The numbers in the column ‘Functional validation’ are references. nd: not determined

### Inactivation of cell cycle and death regulators

In addition to the near universal loss of RB and p53, significant portions of SCLC mutations affect the functional homologs RBL1 (3-4%), RBL2 (5–7%), and TP73 (13%)^20^. RBL1 and RBL2 (also known as p107 and p130, respectively) share key functions with RB, including regulation of the E2F transcription factors in the expression of cell cycle genes. They can compensate for the functional loss of the other RB family members to some extent while exerting unique functions during cell proliferation and differentiation though distinct protein-protein interactions^30–33^. The tumor suppressor activity of these homologs does not appear to be as potent as that of RB in SCLC, as indicated by the dominant selective pressure for RB inactivation. However, it remains poorly understood what roles these RB homologs play in SCLC pathogenesis and why certain subsets of SCLC tumors harbor additional inactivating mutations in RB family members. Similarly, *TP73* encodes tumor protein p73, which, together with the related protein p63, constitutes the p53 family of transcription factors. Because of their structural resemblance to p53, both p73 and p63 are considered tumor suppressors; they indeed control cell cycle arrest and apoptosis via their ability to induce expression of related genes^34–36^. However, unlike ubiquitous expression of p53, p73, and p63 are expressed in a tissue/cell type-specific manner. p73 expression is widespread along the airway epithelium, while p63 expression is limited to basal cells. Notably, *TP73* is frequently altered in the SCLC genome (13%), whereas *TP63* alteration is lacking^20^. The *TP73* alterations include gene rearrangements that result in the generation of variant p73 with NH-terminal truncation (p73Δex2 and p73Δex2/3) or COOH-terminal deletion (p73Δex10). Variants with N-terminal truncation lack the entire transactivation domain or a part of it and may exert a dominant-negative effect on wild-type p73 and p53^37,38^.

### Inactivation of epigenetic regulators

A group of alterations in SCLC appears to converge on genes encoding epigenetic regulators, including CREBBP/EP300 (cAMP response element-binding (CREB)-binding protein/E1A-associated p300), KMT2A and KMT2D (lysine methyltransferase 2A and 2D, respectively), KDM6A (lysine demethylase 6A), and several components of the polybromo-associated BRG/BRM-associated factor (PBAF) complex, including PBRM1 (polybromo 1), ARID1A and ARID2 (AT-rich interactive domain-containing protein 1A and 1D, respectively), and CHD7 (chromodomain-helicase-DNA-binding protein 7)^20–22,39^. CREBBP and EP300 are histone acetyltransferases (HATs) that acetylate several lysine residues on histone proteins. CREBBP/EP300-mediated acetylation of histone H3 lysine 27 (H3K27) results in chromatin structures that are favorable for active transcription of downstream genes^40–43^. While CREBBP/EP300 participate in many physiological processes, including embryonic development, growth control, and homeostasis, by coupling chromatin remodeling to transcription factor recognition, loss of CREBBP/EP300 functions has been implicated in various cancer types, including lymphoma and lung cancer^44,45^. In SCLC, mutations clustered in the HAT domain, as well as gene truncation in the CREBBP and EP300 acetyltransferases suggest a potential role of H3K27 acetylation and the resulting gene activation in tumor suppression. The mutations observed in *CREBBP* and *EP300* were largely mutually exclusive, suggesting a shared tumor suppressor function between these functional paralogs (Fig. 2). KMT2A and KMT2D (also known as mixed lineage leukemia 1 and 2, respectively) are histone methyltransferases specific for histone H3 lysine 4 (H3K4)^46^. KMT2A/2D-mediated monomethylation of histone H3 lysine 4 (H3K4me1) enhances H3K27ac by CREBBP/EP300 at the enhancer/promoter. Gene rearrangements and mutations affecting the SET domains of these proteins have frequently been found in leukemia^47–49^. The missense and truncating mutations in *KMT2A and KMT2D* suggest a role for loss-of-function mutations in these genes, leading to transcriptional repression due to a global reduction in both H3K4me1 and H3K27ac^39,46^. KDM6A demethylates H3K27me3, priming the histone lysine residue for acetylation by CREBBP/EP300. Therefore, both KMT2 family proteins and KDM6A act in concert with HAT proteins to induce and maintain the expression of target genes. In cancer, loss of KMT2 family proteins or KDM6A leads to H3K27 trimethylation (H3K27me3) and silencing of tumor suppressor genes. This H3K27 methylation is driven by PRC2 (polycomb repressive complex 2), a multiprotein enzyme complex composed of EZH2, SUz12, EED, and YY1, and increased activity of EZH2 promotes the development of many cancer types^50^.

**Fig. 3:**
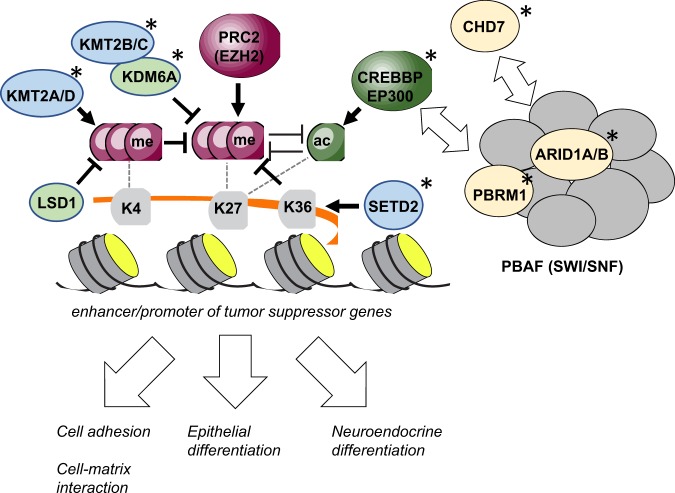
A model of epigenetic regulation in the SCLC genome. This model describes potential interactions among multiple chromatin modifiers on histone marks in the enhancers and promoters of genes. CREBBP/EP300, KMT2 family proteins, and KDM6A act in opposition to PRC2 on H3K27. PBAF complex proteins, CDH7, and SETD2 also participate in the chromatin modification that results in H3K27 acetylation. This epigenetic regulation may influence the expression of numerous genes that are involved in cell-cell and cell-matrix adhesion and epithelial and neuroendocrine differentiation. Asterisks indicate genes recurrently mutated in SCLC. me methyl group, ac acetyl group.

Taken together, a unifying model could emerge to show functional relationships between the chromatin modifiers and the regulatory elements of critical tumor suppressor genes (Fig. 3). CREBBP/EP300, KMT2 family proteins, and KDM6A all oppose the activity of PRC2 by demethylating and acetylating H3K27. When their functions are reduced, these tumor suppressor genes may be vulnerable to PRC2-mediated silencing. Separately, SETD2 may also play a role in preventing this gene silencing because its methylation of H3K36 inhibits the action of PRC2^51^, and SETD2 is significantly mutated in SCLC. While this model provides a simplistic view of epigenetic regulation that is disrupted due to inactivating mutations, it may be expanded to include the components of the PBAF chromatin-modifying complex, whose genes are frequently mutated in SCLC^20,39^. Given the increasing implication of this chromatin-modifying complex in cancer and its interaction with CREBBP/EP300^52^, the defects in PBRM1 and ARID1A/B may contribute to SCLC. Beyond determining the impact of alterations in these epigenetic regulators, defining the functional relationships among them will be important not only for understanding the mechanism of SCLC development but also for identifying synthetic lethality among them^53^. In addition, it will be important to determine target genes that these epigenetic regulators converge on and the upstream factors that control this concerted epigenetic regulation. As discussed below, target genes may play roles in cell homeostasis and maintaining neuroendocrine differentiation and cell adhesion that are often altered during oncogene-driven transformation^54^. The NOTCH pathway may be one of the upstream regulators because it regulates lung neuroendocrine (NE) differentiation and directly interacts with CREBBP/EP300 to activate the expression of target genes^55,56^. Inactivation of either the NOTCH pathway or CREBBP/EP300 may be sufficient to alter NE differentiation during SCLC development, underlying the apparent mutual exclusivity between the recurrent mutations in these genes (Fig. 2)^20,57^.

### Defective networks for cytoskeletal dynamics and cell adhesion

Recent studies identified a group of recurrent mutations affecting genes that are not linked to specific oncogenic pathways, including mutations in ALMS1, ASPM, COBL, COL4A2, COL22A1, FMN2, KIAA1211, PDE4DIP, ROBO1, and SLIT2. Notably, the known functions of these proteins are related to cytoskeleton formation or rearrangements associated with cell–cell and cell–matrix interactions. ALMS1 (Alstrom syndrome gene), ASPM (abnormal spindle-like microcephaly-associated protein), and PDE4DIP (phosphodiesterase 4D interacting protein) bind to microtubules and play a role in the formation of microtubule-based structures, including the centrosome and mitotic spindle^58–60^. One overlapping process on which defects in these genes converge may be cell division. ASPM mutation affects polarity during the cell division of neural progenitor cells and results in microencephaly, and PDE4DIP is a paralog of the microencephaly protein CDK5RAP2^61–64^. COBL (cordon bleu), FMN2 (formin 2), and KIAA1211 (also known as CRAD: cancer-related regulator of actin dynamics) bind to actin and influence actin cytoskeleton dynamics and cell polarity^65–68^. While COBL regulates neuron morphogenesis, including the branching of axons, and has not been implicated in cancer, KIAA1211 is significantly mutated in colorectal cancers, and loss of its function promotes the development of mucinous colorectal cancer in APC-deficient mice. FMN2 is a component of the p14ARF tumor suppressor pathway. SLIT2 (slit guidance ligand 2) and ROBO1 (roundabout guidance receptor 1) are a ligand and a cognate receptor, respectively, that trigger the signaling that controls axon guidance, neurogenesis and cancer progression^69^. While it is not known how this signaling contributes to tumorigenesis, major actin/microtubule cytoskeleton-mediated responses are affected following loss of *SLIT2* or *ROBO1*, resulting in defective cell polarity. Two collagen proteins, COL4A2 and COL22A1, are frequently mutated in SCLC, but it is difficult to link these mutations to any aspect of tumorigenesis. The potential role, if any, of these proteins in the extracellular matrix would be related to stabilizing the cell-matrix interaction by serving as a ligand for cell adhesion proteins, which may be disrupted in transforming cells.

### Alterations of receptor tyrosine kinase pathways

Subsets of lung adenocarcinoma tumors are driven by hyperactive kinase signaling pathways owing to oncogenic alterations in EGFR, ALK, ERBB2, ROS1, and MET. These subsets show clinical responses to several tyrosine kinase inhibitors, including erlotinib and crizotinib. In the SCLC genome, these receptor tyrosine kinases and other kinase signaling mediators, such as KRAS, BRAF, and MEK/ERK, are rarely altered. While most of the genomics studies found few actionable targets in subsets of SCLC tumors, a sequencing study on 98 ‘undifferentiated’ SCLC samples found that 53% of the tumors had at least one actionable alteration^70^. FGFR1 amplification has recently been discovered in a small subset of patient tumors^14,20,21^. While functional studies using SCLC cell lines showed a tumor-suppressive effect with genetic or chemical inhibition of FGFR1^71^, the existing inhibitors are not being tested in SCLC. As the frequency of FGFR1 amplification varies and does not necessarily correlate well with protein expression, and a robust biomarker is needed to predict the response to an FGFR1 inhibitor. MET (also known as c-MET) is another receptor tyrosine kinase occasionally amplified or mutated in SCLC^72–74^. Similar to that of FGFR1 amplification, the frequency of MET alterations varies. Consistent with the deregulation of receptor tyrosine kinase signaling, alterations of intracellular signaling mediators, including IRS2, PIK3CA, AKT, and mTORC1 complex proteins, are detected in SCLC^20,74–76^. Deregulation of the PI3K-mTOR signaling axis may also be achieved by permanent loss of PTEN (phosphatase and tensin homolog), a tumor suppressor and regulator of cell proliferation and migration^15,77^. Indeed, frequent loss-of-function mutations in *PTEN* were found in SCLC, and some mutations are expected to affect the phosphatase activity of the protein^20,21,39^. While all these alterations result in an increase in pathway activity, alterations in EPHA7 (ephrin type-A receptor 7), a member of the ephrin receptor subfamily of tyrosine kinases, cause loss of function. EPHA7 has been implicated in axon guidance and has been shown to play a tumor suppressor role in regulating the growth of lymphoma and prostate tumors^78,79^, but the impact of EPHA7 inactivation on SCLC remains to be determined.

## Models for functional characterization of SCLC genomic alterations

One of the important challenges since the elucidation of the SCLC genome is the paucity of functional information for most of the identified mutations. Functional interrogation of alterations is underway using established SCLC cell line-based models, genetically engineered mouse models, and patient-derived xenograft models. Each of these models has its own strengths and limitations, as briefly discussed below.

### Cell lines and patient-derived tumor models

SCLC cell lines, established primarily from metastatic SCLC tumors, have been a powerful model for characterizing gene function and testing candidate drugs for decades. These cell lines, maintained in culture for decades, are known to acquire de novo alterations that confer a selective advantage to grow in culture conditions but may not be relevant for tumor development and malignant progression in vivo^80,81^. Recent advances in using patient-derived xenograft models based on biopsy/resected tumors (PDX) and circulating tumor cells (CTX) drastically enhance the capacity to identify and test biomarkers for treatment and prognostication^82,83^. Remarkably, these models demonstrate similar pathophysiological features and clinical responses to standard chemotherapy. However, despite the tractability of cell lines and primary tumor cells and their clinical utility in the development of novel therapeutics, these models lack features of premalignancy and are awash with uncharacterized mutations that may be less prevalent at early stages of tumor progression; therefore, they may not be a robust model to systematically characterize genetic alterations for their role in tumor development. It is increasingly clear that functional characterization of the SCLC genome requires an approach integrating patient-derived models and genetically defined mouse models, as well as omics profiling.

### Genetically engineered mice and precancerous cell-based models

**Fig. 4:**
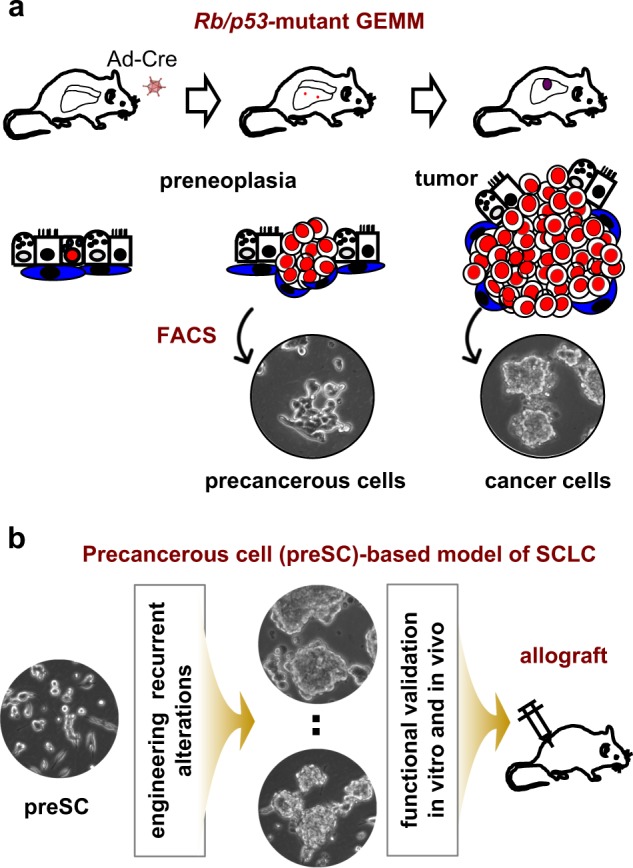
Genetically engineered mouse model and a precancerous cell-based model of SCLC. **a** The *Rb/p53*-mutant GEMM displays a stepwise process of tumor development that includes preneoplasia at early stages. The neuroendocrine tumor cells and precancerous cells (preSCs) can be labeled with a lineage-specific marker and isolated using fluorescence-activated cell sorting (FACS). **b** PreSCs are genetically engineered to mimic patient alterations and tested for tumorigenic potential in multiple in vitro and in vivo assays, including allograft models.

The loss of RB and p53 functions in nearly all SCLC tumors led to the generation of a genetically engineered mouse model (GEMM) in which the mouse orthologous genes *Rb1* and *Trp53* were conditionally deleted using intratracheally instilled adenoviral Cre^29^ (Fig. 4a). The lung tumors that develop in these *Rb*/*p53*-mutant mice recapitulate the histology, neuroendocrine differentiation, metastasis pattern, and even chemotherapy response of human SCLC^29,84^.

This autochthonous model allows for studying early-stage tumor development events under the native gene-environment interactions, which is extremely difficult in patients because the cancer is often detected in late stages, and preneoplastic lesions remain elusive^85^. In addition to its similarity to human SCLC, its relatively long latency makes the *Rb*/*p53*-mutant GEMM a robust model for testing candidate alterations for their role in promoting tumor development^7,86–89^. However, this GEMM is generally limited to testing one candidate at a time and is not ideal for studying gene interactions. Alternatively, *Rb*/*p53*-mutant precancerous neuroendocrine cells serve a streamlined approach to characterize genomic alterations (Fig. 4b). These mutant neuroendocrine cells were isolated from an early-stage lesion in the GEMM using a neuroendocrine lineage-specific green fluorescent protein (GFP) under the control of gene regulatory elements flanking the pan-neuroendocrine gene *Chromogranin A*^90^. Notably, these cells from an early stage of tumor development grow as adherent monolayers in culture and do not readily form subcutaneous tumors in an allograft model, unlike most tumor cells, which aggregate and form spheres and have a high tumorigenic capacity when transplanted into an allograft model. These cells remain nontransformed due to the lack of necessary oncogenic factors and hence were designated the *pre*cancerous cells of *SC*LC (preSC). Their ability to transform upon the introduction of oncogenic alterations alone or in combination makes this preSC model a tractable and robust model for systematic characterization of the SCLC genome^54,90^. Ultimately, to address the complexity of genomic heterogeneity, an integrated approach using multiple models is necessary to facilitate the characterization of a number of mutations alone or in combination. Furthermore, as the synthesis of human and mouse model data led to a draft of SCLC molecular subtypes^6^, functional data from both human and mouse models will help refine the molecular classification and define common or unique vulnerabilities of the subtypes. In the following section, we describe recent findings on a selected group of alterations that were validated using these models.

## Functional validation of recurrent alterations

### PTEN loss drives tumor development

The functional significance of mutant forms of PTEN in SCLC has recently been determined using an *Rb*/*p53*-mutant GEMM^86,87^. Remarkably, inactivation of one allele of *PTEN* was sufficient to accelerate lung tumor development and resulted in frequent metastasis to the liver. These results strongly suggest that PTEN is a critical tumor suppressor in the pathogenesis of SCLC and provide a rationale for treating patients with PTEN mutations with inhibitors of its downstream effectors, PI3K and AKT. Indeed, recent studies showed that the suppression of the PI3K/AKT pathway significantly inhibited the proliferation of SCLC cell lines^86^.

### RBL2 loss promotes tumor development

The recurrent mutations and chromosomal loss of this RB homolog led to the idea that inactivation of this cell cycle regulator is critical for the malignant progression of *RB*-deficient cells. To test this idea, Schaffer et al. used a variant of the SCLC GEMM in which *Rbl2* was deleted in combination with *Rb1* and *Trp53* in adult lung epithelial cells^88^. The mice with *Rb1*, *p53*, and *Rbl2* deletions (*Rb*/p53/*Rbl2* mice) developed many more tumors than those with only *Rb* and *p53* deletions (*Rb*/p53 mice); these drastic changes in tumor incidence and latency clearly indicate that Rbl2 is a potent tumor suppressor in SCLC development. A genome-wide expression profiling experiment showed that *Rb*/*p53*/*Rbl2*-mutant mouse tumors were very similar to *Rb*/*p53*-mutant tumors. The histopathological features of *Rb*/p53/*Rbl2*-mutant tumors also resembled those of human SCLC, while more tractable features, including a relatively short latency, make this *Rb*/*p53*/*p130*-mutant model useful for studying the mechanisms of SCLC initiation, progression, metastasis, and tumor immunity and a novel preclinical mouse model for testing new therapeutics against SCLC^91–95^.

### The MYC family members in different subtypes of SCLC

Since the discovery of recurrent amplification among the MYC family genes in the 1980s, a number of reports implicated these proto-oncogenes in SCLC but lacked functional evidence for their roles in multiple aspects of SCLC tumorigenesis until recently. Recently, Kim et al. demonstrated retroviral L-Myc-driven transformation of precancerous cells into malignant tumor cells^90^, and Hujibers et al. showed that L-Myc expression in the *Rb*/*p53*-mutant GEMM increased lung tumor development^96^. These studies provide critical evidence for the potent oncogenic role of L-Myc decades after its discovery in SCLC^19,90,96^. Kim et al. also showed that deletion of *Mycl1* (encoding L-Myc) reduced tumor incidence and burden in the lungs of both the *Rb*/*p53*/*p130* and *Rb*/*p53*/*Pten*-variant models of SCLC. Furthermore, a transcriptome analysis comparing L-Myc-amplified preSC with control preSC models indicated enrichment of ribosome biogenesis and protein translation among the most significantly altered genes during oncogene-driven transformation. The significance of these changes was demonstrated when inactivation of ribosome biogenesis via chemical inhibition of RNA Pol I-driven ribosomal RNAs suppressed tumor growth in both the *Rb*/*p53*-mutant and *Rb*/*p53*/*p130*-mutant GEMMs.

Likewise, Mollaoglu et al. determined the impact of hyperactive c-MYC, which results from frequent gene amplification or overexpression, using a variant of the SCLC GEMM^7^. In this model, conditional expression of a hyperactive form of c-Myc (Myc^T58A^) in *Rb* and *p53*-deficient lung epithelial cells accelerated tumor development and resulted in tumors with a higher capacity for proliferation and metastasis than those with expression of a variant of c-MYC. Notably, the pathology of c-Myc-driven tumors resembled that of an SCLC subtype known as ‘variant’^9^. This c-Myc-driven tumor derived from both the GEMM and human tumors (recently named SCLC-N) tends to express high levels of NEUROD1 (neuronal differentiation 1; a master transcriptional regulator of neural development) and low levels of neuroendocrine markers, in contrast with L-MYC-driven tumors (recently named the SCLC-A subtype), which express high levels of both ASCL1 (achaete-scute homolog 1; another regulator of neural differentiation) and neuroendocrine markers, including SYP and CGRP^6,9,20,97–99^. Given the stage-specific distinct roles of ASCL1 and NEUROD1, it is tempting to speculate that these two subtypes originate from neuroendocrine lineage cells at different stages of differentiation. Alternatively, each of the MYC family proteins determines subtype-specific phenotypes, and current data indicate this may be the case. Tumors in *Rb*/*p53*/*Myc*^*T58A*^ mice change from the ASCL1-positive/NEUROD1-negative subtype to the ASCL1-negative/NEUROD1-positive variant subtype with loss of NE cell markers^7^. Furthermore, the tumors obtained from Myc-driven mouse SCLC and patient tumors with a high level of MYC showed selective sensitivity to the inhibition of Aurora kinase^7,100^, suggesting Aurora kinases as a molecular vulnerability in SCLC cells with MYC family amplification. Similarly, inactivation of the MYC binding protein MAX in MYC-high SCLC caused synthetic lethality with BRG1 inhibition^101^, and inhibition of arginine synthesis with pegylated arginine deiminase (ADI-PEG-20) selectively suppressed the growth of MYC-driven tumors in GEMMs, human cell lines, and patient-derived xenografts from a relapsed patient^102^. These findings from the study of the GEMMs and the novel preSC-based model helped identify molecular subtypes of MYC family-driven SCLC, adding to the understanding of the oncogenic mechanisms and therapeutic vulnerabilities for each cancer subtype.

### Increased NFIB activity promotes tumor development and metastasis

Copy number amplification at the *NFIB* locus and protein overexpression in the metastatic tumors in the GEMM and human cell lines derived from metastasis prompted multiple investigations into a role for NFIB in SCLC^21,27,28,103,104^. Denny et al. showed that increased NFIB was sufficient to disseminate tumor cells partly by altering a genome-wide increase in chromatin accessibility at distal regulatory elements for a large number of genes functionally associated with neural development, cell adhesion, and motility. Semenova et al. also supported the role of increased NFIB in promoting metastasis by showing that transcription-driven metastatic spread correlated well with a poor differentiation status and the E-CADHERIN (CDH1)-negative-related invasiveness of tumor cells^27^. Similarly, Wu et al. also found that NFIB amplification and overexpression are far more frequent in liver metastases than in primary tumors and that gene overexpression accelerated lung tumor development in the *Rb*/*p53*-mutant GEMM before metastatic spread^28^. In addition, these studies consistently showed that NFIB overexpression increased cell viability and proliferation during transformation and that suppression of NFIB expression in cell lines inhibited cell proliferation and activated cell death. However, although this NFIB-driven transcription program promotes tumor growth and metastasis, it confers cisplatin sensitivity to tumors that are otherwise refractory to chemotherapy^105^. These findings strongly suggest that increased transcriptional activity of NFIB is a key driver of SCLC development and malignant progression but may introduce a vulnerability to be exploited for therapeutic intervention.

### Inactivation of CREBBP promotes tumor development through altering cell adhesion

The functional significance of recurrent mutations in *CREBBP* has been validated using the *Rb*/*p53*-mutant GEMM, as well as the precancerous cell (preSC)-based model^54^. Jia et al. showed that CRISPR-mediated targeting of the HAT domain in CREBBP transformed preSCs into malignant SCLC cells. Furthermore, complete loss of CREBBP also accelerated tumor development in the autochthonous mouse model and significantly reduced overall animal survival. This study found from comparative gene expression analyses that CREBBP inactivation results in reduced expression of tight junction and cell adhesion genes, including CLAUDINs and E-CADHERIN (CDH1). These genes, particularly CDH1, function to maintain epithelial integrity and suppress transformation. In support of this idea, CDH1 knockout increases the capacity for anchorage-independent growth and colony formation from single cells, and restoring CDH1 expression inhibited these capacities in CREBBP-mutant cells. These findings suggest that the CREBBP/EP300-CDH1 axis of the tumor suppressor pathway is frequently inactivated in SCLC. Furthermore, the study found that CREBBP-mediated histone acetylation directly activates the regulatory elements of *CDH1* and other adhesion-related genes and opposes the influence of histone deacetylases (HDACs). Importantly, CREBBP-deficient SCLC exhibited exceptional responses to pracinostat, which is known to inhibit HDACs in vivo.

### Roles of the NOTCH pathway in tumor heterogeneity

The recurrent mutations affecting the NOTCH family members strongly indicate a tumor suppressor function of the pathway that is known to negatively regulate neuroendocrine (NE) differentiation during lung development^20,57^. A previous study showed that ectopic expression of N1ICD (Notch1 intracellular domain) in both mouse and human SCLC cell lines inhibited their proliferation by inhibiting cell cycle progression^106^. Similarly, George et al expressed an intracellular domain of Notch2 (N2ICD; an active form of the Notch receptor) in the *Rb*/*p53*/*Rbl2*-mutant cells of a GEMM and found a significant reduction in lung tumor development and the growth inhibition of SCLC cell lines^20^. However, using a knock-in GFP reporter for the NOTCH pathway activity incorporated into the SCLC GEMM, Lim et al. found that the NOTCH pathway-active/GFP-positive cells coexisted with the NOTCH pathway-inactive/GFP-negative tumor cells in the lung tumor^107^. Intriguingly, the NOTCH pathway-inactive tumor cells following stimulation with DLL4 (a delta-like ligand 4) gave rise to NOTCH pathway-active cells lacking NE markers. This NOTCH pathway-driven switch to non-NE cells occurred in 10–50% of NE cells and was mediated in part by REST (RE1-silencing transcription factor), a transcriptional repressor of neural differentiation and a direct target of the NOTCH pathway. From multiple in vitro and in vivo assays, this study concluded that the transdifferentiated non-NE cells were relatively resistant to the standard chemotherapy drugs (carboplatin and irinotecan) at doses that effectively killed NE tumor cells. The heterogeneity in SCLC was noted in the cancer cell lines and has recently been observed in the mouse model^108^, although the cellular and molecular origins of heterogeneity have been unclear. This tumor cell-driven creation of intratumor heterogeneity has also been observed in lung adenocarcinoma. In KRAS-driven tumors, the niche cells derived from tumor cells support the tumor cells by secreting WNT ligands in a paracrine manner^109^. This idea that tumor cells generate their own niche cells is provocative but may be important in approaching the problem of intratumoral heterogeneity associated with chemotherapy^82,83^. In line with that, a combination of the standard chemotherapy (carboplatin plus irinotecan) and NOTCH inhibition (tarextumab) showed greater efficacy than the chemotherapy alone. The result of this preclinical trial suggests that inhibition of the Notch pathway in combination with chemotherapy may be more efficacious in preventing early-stage SCLC progression and relapse following existing chemotherapies. While this idea of combination therapy needs to be further tested, questions remain in regards to the relationship between the NOTCH pathway and other oncogenic pathways and to cell plasticity and its molecular determinant. NOTCH pathway-driven regulation of ASCL1 and MYCL1 may help link NOTCH pathway alterations to one of the emerging molecular subtypes, especially SCLC-A. Interestingly, pharmacological inhibition of LSD1 (lysine-specific histone demethylase 1, also known as KDM1A) suppressed tumor growth in a chemoresistant PDX model, and it coincided with activation of the NOTCH-REST axis and downregulation of ASCL1^110^. As LSD1 inhibits KMT2D, this finding suggests a role for the epigenetic regulation of NOTCH expression and supports the concept of inhibiting LSD1 as a new subtype-specific therapy.

## Conclusion and outstanding questions

Thanks to advances in omics approaches and disease models, there has been remarkable progress in the field of SCLC over the past few years. Comprehensive profiling of the SCLC genome has uncovered a list of candidate oncogenic drivers for this once enigmatic cancer. Extensive efforts are underway to determine the functional impact and significance of all significant alterations. The functional validation of a small list of genomic alterations in multiple models as described above has already provided critical insights into the cellular and molecular mechanisms of SCLC development and a glimpse into the molecular heterogeneity underlying SCLC. As a functional map of the genomic landscape in SCLC will be established in the near future, the challenges ahead include gaining a mechanistic understanding of the molecular networks involving the characterized alterations and developing tumor models that more accurately reflect patient-specific sets of alterations. Ultimately, advances in defining the SCLC genome will pave the way for the discovery of the common and specific mechanisms, as well as vulnerabilities associated with the genomic alterations.
